# Evaluation of rhizomania infection on sugar beet quality in multi‐year field assessment

**DOI:** 10.1002/fsn3.4069

**Published:** 2024-04-05

**Authors:** Parviz Fasahat, Mohsen Aghaeezadeh, Dariush Taleghani, Mozhdeh Kakueinezhad, Mostafa Hosseinpour, Rosa Angela Pacheco

**Affiliations:** ^1^ Sugar Beet Seed Institute (SBSI) Agricultural Research, Education and Extension Organization (AREEO) Karaj Iran; ^2^ Biometrics and Statistics Unit International Maize and Wheat Improvement Center (CIMMYT) Mexico Mexico

**Keywords:** genotype by environment, impurity, quality, rhizomania, sugar beet

## Abstract

Rhizomania is one of the most destructive and damaging sugar beet diseases that has spread in different regions of Iran. In order to evaluate the genotypic, environmental, and genotype by environmental variability of sugar beet genotypes under rhizomania infection, variance components were estimated from the trial series in 7 years. Required data, such as yield and quality parameters, were collected from value for cultivation and use trials. Results of analysis of variance showed that the environment was the source that explained most of the variability, except for amino‐N and alkalinity. Quality traits were also influenced by the environment × cultivar interaction, so that 4.8% (white sugar content) to 46.1% (alkalinity) variance was observed. In contrast, genetic variation was much lower, between 1.2% (potassium) and 27.4% (amino‐N). A strong and negative correlation was found between root yield, sugar yield, and white sugar content with the disease index, which obviously illustrates the negative impact of the rhizomania on root weight and as a consequence on the dependent traits. The cluster analysis of the cultivars based on the quantitative and qualitative traits and the disease index showed that the range of variation in traits, such as the disease index, varied from 6.25 for the susceptible cultivar to 1.25 for the resistant one. This indicates the existence of sufficient genetic diversity among cultivars in terms of this trait. High impurity accumulation was observed in Shiraz region compared with Mashhad. In conclusion, it is observed that rhizomania has a significant effect on the impurity concentration in the root, especially sodium, potassium, and amino‐N. This is very important in the sugar industry because sugar extraction depends on the concentration of these impurities, in addition to the sugar content of each cultivar.

## Graphical abstract

Rhizomania is one of the most devastating diseases of sugar beet. In a 7‐year field assessment, the effect of field infection on rhizomania was evaluated. Results showed that the disease affects the root quality remarkably, which may assist plant breeders in the development of resistant cultivars.

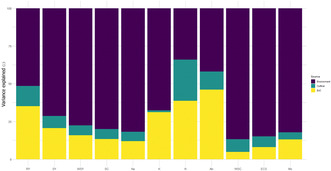

## 1 INTRODUCTION

Sugar beet (*Beta vulgaris*), as one of the two most important sources of sugar supply in the world and with a global cultivated area of 4.5 million hectares, has been allocated about 105,000 hectares of arable land in Iran, which is equivalent to 0.92% of all crops and 24.3% of the entire harvest level of industrial products (Amiri et al., 2023; FAO, 2022).

The sugar beet plant has constantly been attacked by various pests and diseases. Rhizomania disease is still a problem in many areas of sugar beet cultivation in Iran and throughout the world. The cause of the disease is beet necrotic yellow vein virus (BNYVV), which is inoculated and carried by the fungus *Polymyxa betae* (Canova, 1959; Nusayr, 2024; Tamada & Baba, 1973). The continuation of sugar beet cultivation and the economic life of the sugar industry are largely and undeniably dependent on resistance to rhizomania disease. The disease results in irreparable damage to yield and quality of the sugar beet (Ilbagı et al., 2024; Pavli et al., 2011). Due to the severe reduction in the crop yield and almost unlimited durability of the disease agent in infected soil, as well as the arduousness of its control, rhizomania became as the limiting factor of sugar beet cultivation and, consequently the sugar industry in Europe. In addition to severe reduction in root weight, the disease also induces a reduction in the amount of sugar, so that the sugar yield per hectare is reduced to half or less. If the infection occurs early in the growing season, the extent of damage to the root increases significantly, and thus the production efficiency and the sugar content decrease. The final production potential of the crop under rhizomania infection depends mainly on the cultivar resistance and weather condition (Asher, 2002; Henry, 1996; Khorshidpour et al., 2020).

Rhizomania disease was first reported from sugar beet fields in the southern part of the country in Fars province (Izadpanah et al., 1996). Since then, the wide spread of the disease has been reported in other provinces. The first evaluation on the rhizomania outbreak was conducted in 2001 to 2003 on 3972 root samples collected from 103 fields, and the highest infection was reported in Khorasan province, followed by Fars, Isfahan, Zanjan, Qazvin, Semnan, Kermanshah, West Azerbaijan, and Hamedan, respectively (Farzadfar et al., 2008). The first evaluation indicated the prevalence of type A of the virus; however, later research confirmed the presence of type B of the virus in some areas of sugar beet cultivation. In the following years, the presence of type P of the virus was reported in the sugar beet fields of Razavi Khorasan, North Khorasan, Ardabil, West Azerbaijan, and Kermanshah provinces (Mehrvar et al., 2009).

It is known that the disease can be controlled with some fumigating pesticides, such as methyl bromide or dichloropropene. This measure has been effective in controlling the disease vector and increasing the crop yield from 1.3 to 7.3 t/ha in the USA (Martin & Whitney, 1990) and from 2.1 to 6.9 t/ha in France (Richard‐Molard, 1985). Regardless of this remarkable effect, using this type of chemical is not economically justified and is against the environment. Therefore, other alternative methods should be considered to control the disease. Biological methods using bacterial and fungal agents to prevent the colonization of sugar beet roots by *P. betae* have also been used. Results showed that the bacterium *Pseudomonas* fluorescence, which was used as a seed treatment, cannot control the disease (Resca et al., 2001). However, various types of *Trichoderma* species can reduce rhizomania outbreak between 21 and 68% (Jakubikova et al., 2006). Determining the effectiveness of each method in preventing or slowing down the disease widespread is also very difficult (Asher, 2002). Considering these problems, sugar beet factories and producers decided to use other control strategies, one of which was resistant cultivars. First measures to identify the resistance source to rhizomania in sugar beet started in 1970, which took 10 years to report the resistance source (Biancardi et al., 2002; Tamada et al., 2016). In the USA, the Holly Sugar Company was looking for a variety resistant to the virus and thus introduced the variety Holly. This resistance was provided as a dominant resistance with the *Rz1* gene (Scholten et al., 1996). Plants with this gene showed a very high resistance, but some observations illustrated that under severe infection, the resistance with only one dominant gene could not be sufficient. Later, it was found that cultivars with both *Rz1* and *Rz2* genes were less infected with BNYVV isolates (Liu & Lewellen, 2007). If the sources of permanent resistance are unavailable, other methods, including the production of resistant transgenic plants, should be used to create resistance against the virus.

Before the release or commercialization of a crop, plant breeders evaluate new varieties in several regions for several years. After analyzing the results, the breeder can identify and introduce high‐potential varieties. The purpose of these trials is to ensure the adaptability of the new varieties in the areas where the breeder intends to introduce his variety. If the promising varieties illustrate suitable agronomic value for use in the tested areas, these varieties will be added to the national list of agricultural cultivars. Based on the performance results of the new cultivars, growers select and cultivate the desired one. The aim of this study was to evaluate the effect of genotype and environment on a variation of yield and quality traits of sugar beet cultivars under rhizomania infection over multi‐year trials.

## 2 MATERIALS AND METHODS

### 2.1 Data collection

Yield data were collected from the official sugar beet variety trials, value for cultivation and use, conducted by the Sugar Beet Seed Institute (SBSI) from 2015 to 2021. The total dataset comprised of 54 experiments, in which 77 commercially registered cultivars were evaluated in fields infected with rhizomania in two locations of Mashhad and Shiraz (Table 1, Table S1). All trials were performed in randomized complete block design with four replications. Seeds were planted in early April, and roots were harvested from October to November.

**TABLE 1:** Salient features of the studied sites.

Site	Altitude (m)	Longitude	Latitude	Soil type
Mashhad	998	60°48′E	35°12′N	Silty‐loam
Shiraz	1598	52°42′E	29°46′N	Clay‐loam

At harvest, roots were weighted, cleaned, and quality traits were measured from pulp samples taken from the root at the Sugar Technology Laboratory in SBSI. Sodium, potassium, amino‐N, and sugar content were measured using Betalyser (Anton Paar, Germany) automatic beet laboratory system. Other quality traits were calculated using the following equations (Cooke & Scott, 2012; Reinefeld et al., 1974):

(1) SY=RY×SC

(2) WSY=RY×WSC

(3) $$\mathrm{ECS}=\left(\mathrm{WSC}/\mathrm{SC}\right)\times 100$$

(4) $$\mathrm{MS}=0.343\;\left(\mathrm{Na}+K\right)+0.094\;\left(\text{amino}-N\right)–0.31$$

(5) WSC=SC–MS

(6) $$\mathrm{ALC}=\left(\mathrm{Na}+K\right)/\text{amino}-N$$

In these equations, SY is sugar yield (t/ha), RY is root yield (t/ha), SC is sugar content (%), WSY is white sugar yield (t/ha), WSC is white sugar content (%), ESC is extraction coefficient of sugar (%), MS is molasses sugar (%), Na^+^ is sodium (meq.100/g), K^+^ is potassium (meq.100/g), amino‐N is nitrogen (meq.100/g), and ALC is alkalinity (%).

### 2.2 Disease resistance of the studied cultivars

**FIGURE 1:**
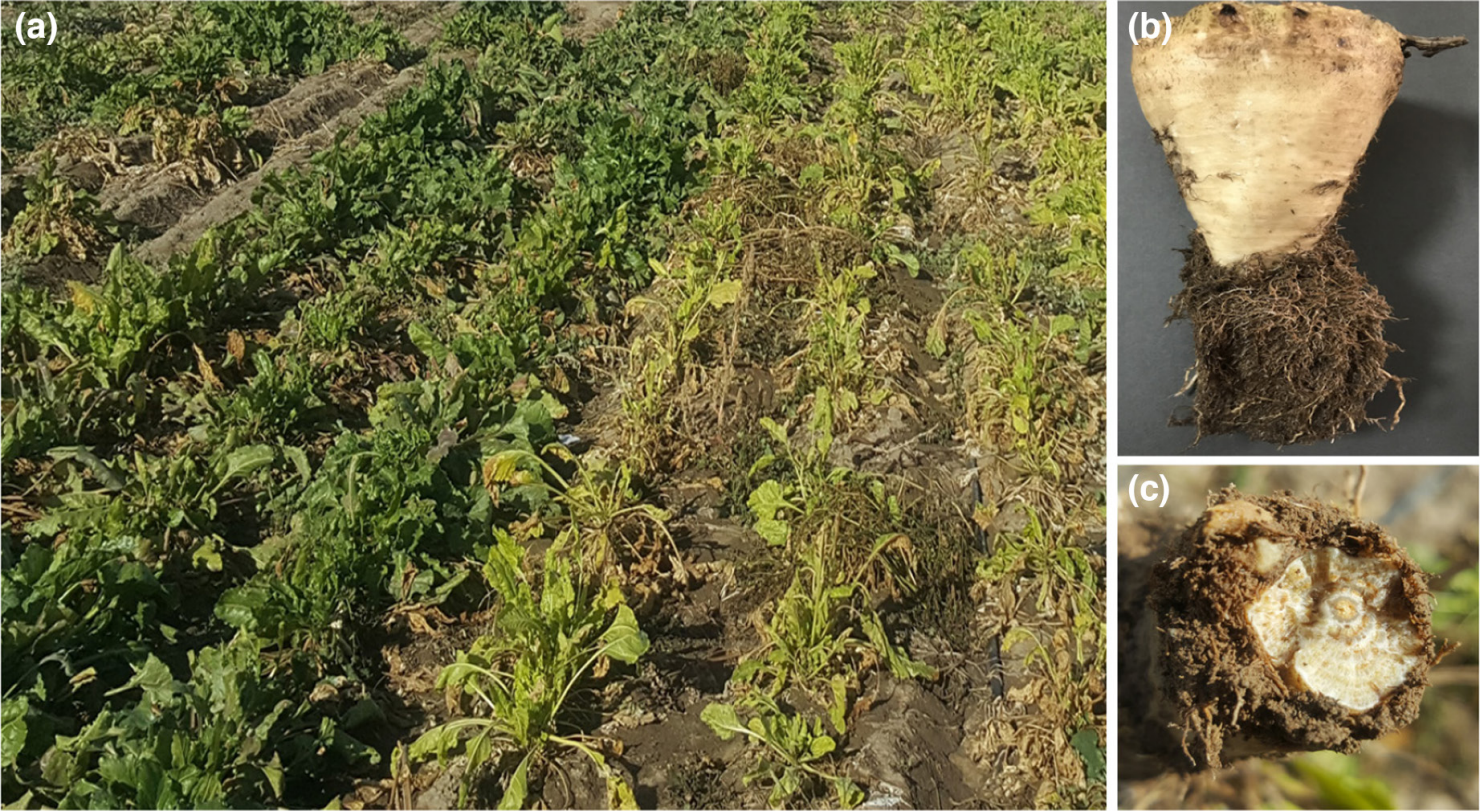
Symptoms of rhizomania on sugar beet plant: (a) the foliage turns into yellow color in susceptible cultivar, (b) development of lateral roots, and (c) necrosis of the vascular ring (photographs taken by Mozhdeh Kakueinezhad).

Resistance to rhizomania disease was evaluated, based on scores given according to the scale of 1 to 9 (Luterbacher et al., 2005). Scores were given to the roots of the plot and not to the individual roots. Our results in years of conducting trials in infected soil to rhizomania showed that the roo of susceptible cultivars becomes necrotic and had many secondary roots, with low volume and weight (Figure 1). These symptoms were not observed in resistant cultivars. Under healthy soil conditions, roots of all studied cultivars were normal. Under infected conditions, due to the destruction of the vascular system, the sugar beet root cannot play its role in supplying water and nutrients needed for plant growth. Therefore, all plant organs are affected by the disease, and the rate of root and leaf growth decrease (Keller et al., 1989).

### 2.3 Statistical analysis

Since new cultivars were tested for 2 years and after that were withdrawn, the datasets were nonorthogonal. Each year, the same set of cultivars was evaluated in different locations. The data were balanced over the years with respect to cultivars and locations. The data obtained from the cultivars in different experimental periods were discrete, except for a few controls cultivated in several periods.

To obtain the adjusted means and variance components for the studied variables of interest, we adjusted for each one the model:

$$\begin{array}{c} y_{\text{ijklm}}=\mu +{\text{Block}}_{i}+{\text{Trial}}_{j}\left({\text{Block}}_{i}\right)+{\text{Year}}_{k}+{\text{Location}}_{l}+{\text{Cultivar}}_{m}\\ +{\text{Location}}_{l}\times {\text{Cultivar}}_{m}+\varepsilon_{\text{ijklm}} \end{array}$$

where $y_{\text{ijklm}}$ is the trait of interest, μ is the mean effect, ${\text{Block}}_{i}$ is the effect of the *i*th block, ${\text{Trial}}_{j}\left({\text{Block}}_{i}\right)$ is the effect of the *i*th block within the *j*th trial, ${\text{Year}}_{k}$ is the effect of *k*th year, ${\text{Location}}_{l}$ is the random effect of *l*th location, ${\text{Cultivar}}_{m}$ is the random effect of *m*th cultivar, ${\text{Location}}_{l}\times {\text{Cultivar}}_{m}$ is the random effect of interaction, and $\varepsilon_{\text{ijklm}}$ is the error that is assumed to be normally and independently distributed, with mean zero and homoscedastic variance $\sigma^{2}$.

With the adjusted means obtained, we proceeded to calculate the phenotypic correlations between variables of interest, for which Pearson's simple correlation was used.

The least significant difference (LSD) with a 5% level of significance was calculated to make the comparisons by pairs of means, and thus it was determined if for each variable of interest, there were significant differences between locations.

$$\mathrm{LSD}=t_{\left(1-0.05,{\mathrm{df}}_{\text{error}}\right)}\times \text{ASED}$$

where $t_{\left(1-0.05,{\mathrm{df}}_{\text{error}}\right)}$ is the cumulative Student's t distribution, 0.05 is the selected level of signficance to 5%, ${\mathrm{df}}_{\text{error}}$ is the degrees of freedom for variance of error, and ASED is the average standard error of the differences between pairs of means. All statistical analyses were performed using R Statistical software 4.0.3.

## 3 RESULTS AND DISCUSSION

### 3.1 Phenotypic data description

The distribution of the phenotypic values of the observed traits showed that all traits had almost a normal distribution throughout the experimental environments, which fulfills the assumption of normality in classical statistical methods. We observed a range in trait differential from low‐performance cultivars to high‐performance cultivars. Normality plots showed heterogeneity of variability for observed traits across environments, indicating the presence of genotype–environment interaction (GEI).

### 3.2 Variance components

**FIGURE 2:**
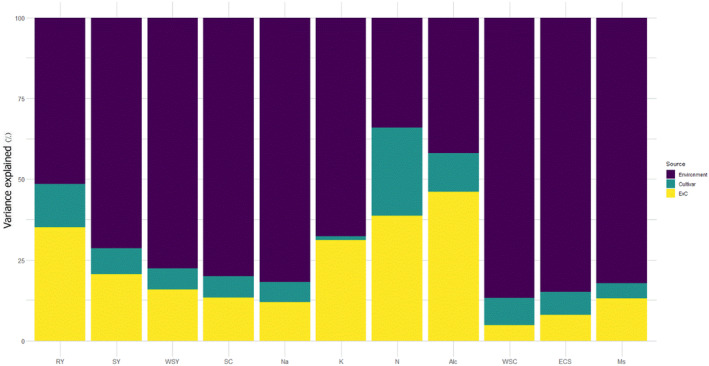
Source of variation of all traits within studied genotypes. The colors represent the source of variation (environment, cultivar, and E × C). Response variables are shown on the *X*‐axis. The proportion explained by each source of variation for each response variable is observed on the *Y*‐axis (percentage). RY, root yield; SY, sugar yield; WSY, white sugar yield; SC, sugar content; Na, sodium; K, potassium; N, nitrogen; ALC, alkalinity, WSC, white sugar content; ESC, extraction coefficient of sugar; MS, molasses sugar.

After adjusting a mixed model where we include block, year (Y), and trial (block) as fixed effects and location, cultivar, location × cultivar (L × C) as random effects, it was observed that location, cultivar, and their interaction had a significant effect (*p* < .05). The analysis of the components of variance (Figure 2) evidenced that in almost all variables, the environment (L × Y) was the source that explained the most considerable variability, except for variables like amino‐N and alkalinity. Such environmental effects are in congruence with the results of Bakare et al. (2022), who also reported 48.6–63.9% of the phenotypic variance by environment as the largest source of variation. Higher environmental variance for main quality traits, such as sugar yield, white sugar yield, sugar content, and extraction coefficient of sugar, indicated that these are the most variable traits and are dependent on inherited genetic effects and the location. Since white sugar content is an estimate of sugar content and molasses sugar, a high environmental effect (86.8%) was observed. This is in accordance with the earlier studies of Hoffmann et al. (2009), who accounted the environment for about 80% of the total variance for all yield and quality parameters of sugar beet. The influence of cultivar, environment, and E × C interaction on components of variance for sugar yield is of a similar pattern as that of white sugar yield: low components of variance for cultivar, but relatively large variance for environment followed by E × C interaction. Sodium showed low E × C interaction (11.9%), which revealed that its accumulation in the root is mainly determined by the environment and genetic diversity. Sodium and potassium contents are mostly determined by local growing conditions during the growing season, whereas sugar content is not only influenced by the agronomic practice but also by the postharvest condition. The relatively high environmental impact on sodium, potassium, sugar content, and molasses sugar is in agreement with that reported in Hoffmann et al. (2009) and with the results from a meta‐analysis of Fasahat et al. (2020). Variation in root yield was not as strongly influenced by environment compared with other main quality traits. This may be explained by the fact that root yield is controlled by nonadditive genes and has high heritability (Fasahat et al., 2018, 2021, 2022).

The cultivar had the lowest role in potassium content by explaining approximately 1.2% of the variation. In a two‐year study by Darabi et al. (2017), the response of commercially resistant cultivars under rhizomania‐infected condition in Shiraz was different regarding potassium content. The average potassium content decreased in resistant cultivars, but increased in other cultivars. Environment × cultivar interaction was responsible for 46.1% of the variance in alkalinity. Compared with the result of this study, Hosseinian et al. (2019) reported a higher impact of genotype on the extraction coefficient of sugar and alkalinity. In a study by Laidig et al. (2017), 30% of the variance components for grain yield in winter rye was allocated to the location with genotype contributed minor influence (2%) on total variation. They have also reported low variation due to L × C interaction. However, Tassinari et al. (2022) reported a 20% variation in the weight of 100 berries in grapevine by the cultivar.

### 3.3 Correlation of root yield and quality traits

**FIGURE 3:**
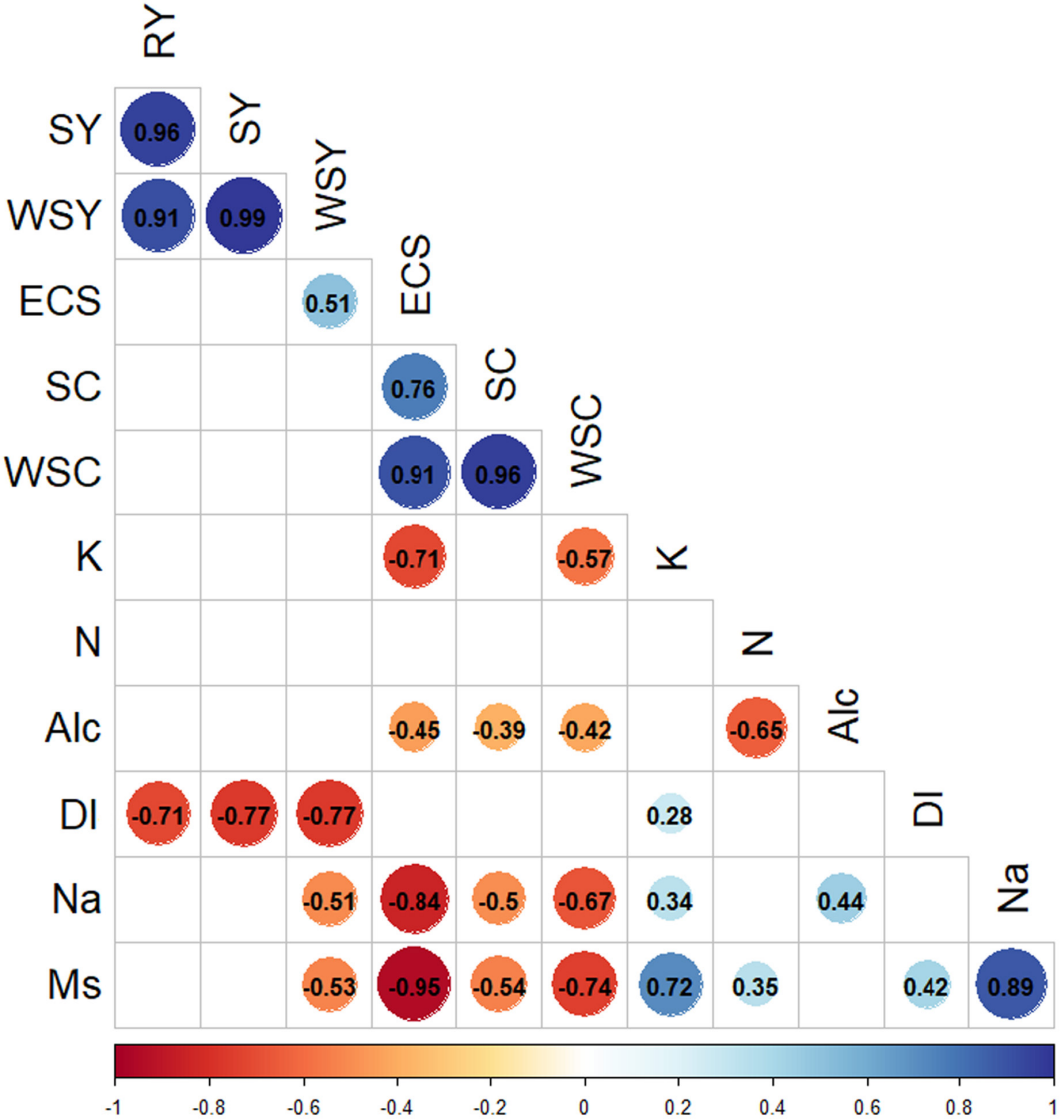
Relationship among sugar beet traits. RY, root yield; SY, sugar yield; WSY, white sugar yield; SC, sugar content; Na, sodium; K, potassium; N, nitrogen; ALC, alkalinity, WSC, white sugar content; ESC, extraction coefficient of sugar; MS, molasses sugar; DI, disease index.

According to the results derived from the Spearman correlation matrix (Figure 3), positive and highly significant correlations were observed between root yield, sugar yield, white sugar yield, sugar content, white sugar content, and extraction coefficient of sugar. However, the above‐mentioned traits showed a negative correlation with sodium, potassium, amino‐N, and molasses sugar, as also reported by Heijbroek (1989), Tuitert (1994), and Ahmadi et al. (2022), under rhizomania stress. Sodium like potassium reduces the purity of syrup and increases sugar waste in the form of molasses. Nitrogenous substances, especially amino acids, also play a significant role in creating molasses, and for this reason, they are called harmful nitrogen. The most important harmful nitrogen compounds are amino acids and betaine, which are not separated in the purification steps and enter the molasses. Increasing the technological quality of the sugar beet crop can be achieved by increasing the sugar content and reducing nonsugar substances, including potassium, sodium, and amino‐N; these impurities not only prevent the crystallization of sucrose, but also reduce sugar extraction ability and increase the amount of molasses. A strong and negative correlation was found between root yield, sugar yield, and white sugar content with the disease index, which obviously illustrates the negative impact of the rhizomania on root weight and as a consequence on the dependent traits to it.

### 3.4 Cluster analysis

**FIGURE 4:**
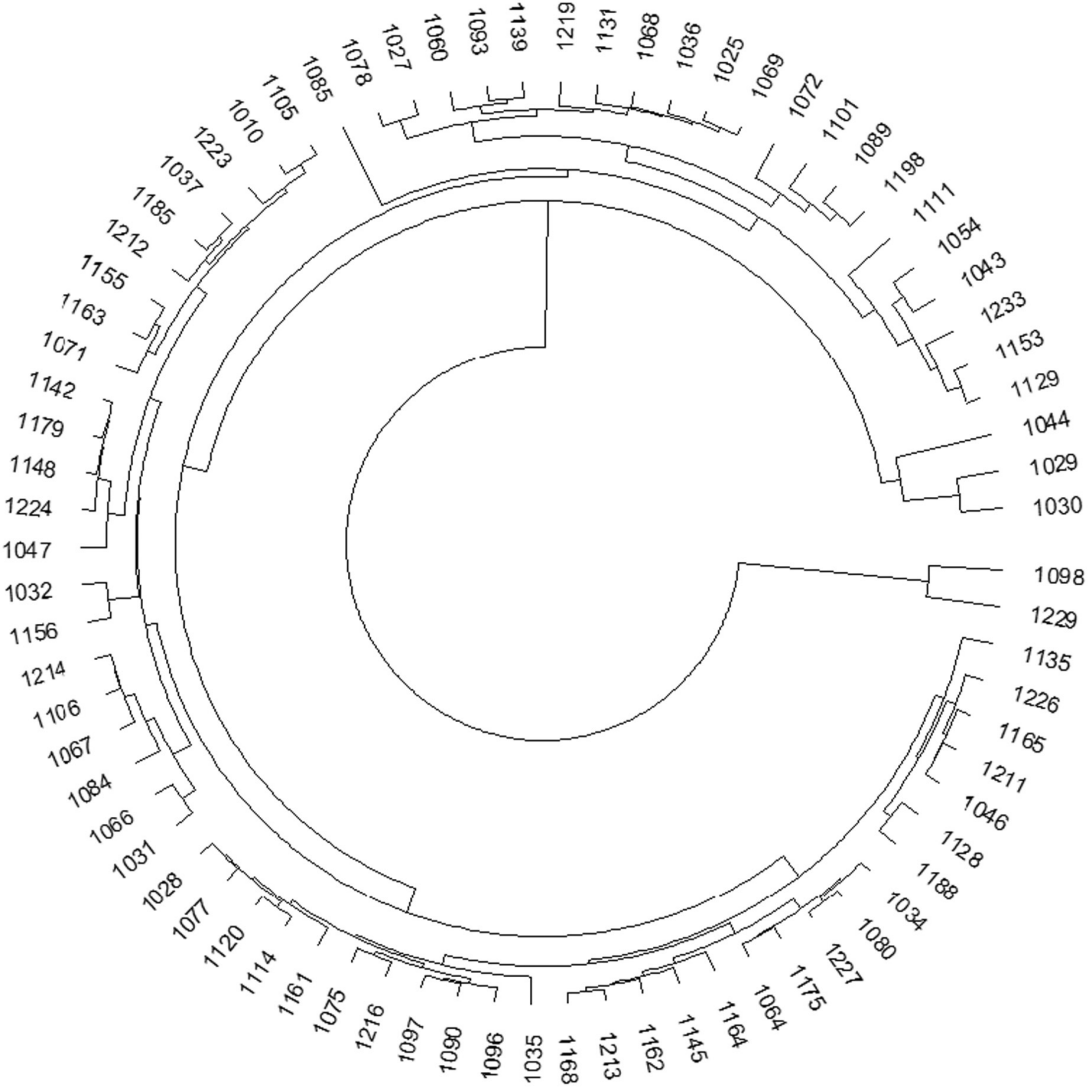
The cluster analysis of the cultivars based on the quantitative and qualitative traits and the disease index.

The cluster analysis of the cultivars based on the quantitative and qualitative traits and also the disease index showed that the range of variation in traits such as the disease index varied from 6.25 for the susceptible cultivar (1114) to 1.25 for the resistant one, 1027 (Figure 4). This indicates the existence of sufficient genetic diversity among cultivars in terms of this trait. Resistant and susceptible cultivars formed a visibly distinct cluster in the dendrogram. Other cultivars that were less resistant were classified as semi‐resistant in separate clusters. Out of 77 cultivars, about 66% were classified as resistant, 31% as semi‐resistant, and 3% as susceptible to rhizomania. Cultivars 1027, 1072, 1029, 1071, 1075, 1068, and 1031 showed remarkable resistance to rhizomania disease. Since other cultivars were classified in the same cluster with the resistant cultivars in the dendrogram, it can be concluded that the resistance created by *RZ*_*1*_ genes is not the same in different genotypes and several factors affect the expression of these genes (Darabi et al., 2017). Owing to the high intensity of field infection in Mashhad and Shiraz, the results of cultivars' evaluation under normal condition were able to have enough credit to confirm the breeding activities of resistant cultivar development. Since the disease index had a negative correlation with the final yield of the crop, it can be concluded that the field screening results of rhizomania resistance sources can be valid and reliable.

**FIGURE 5:**
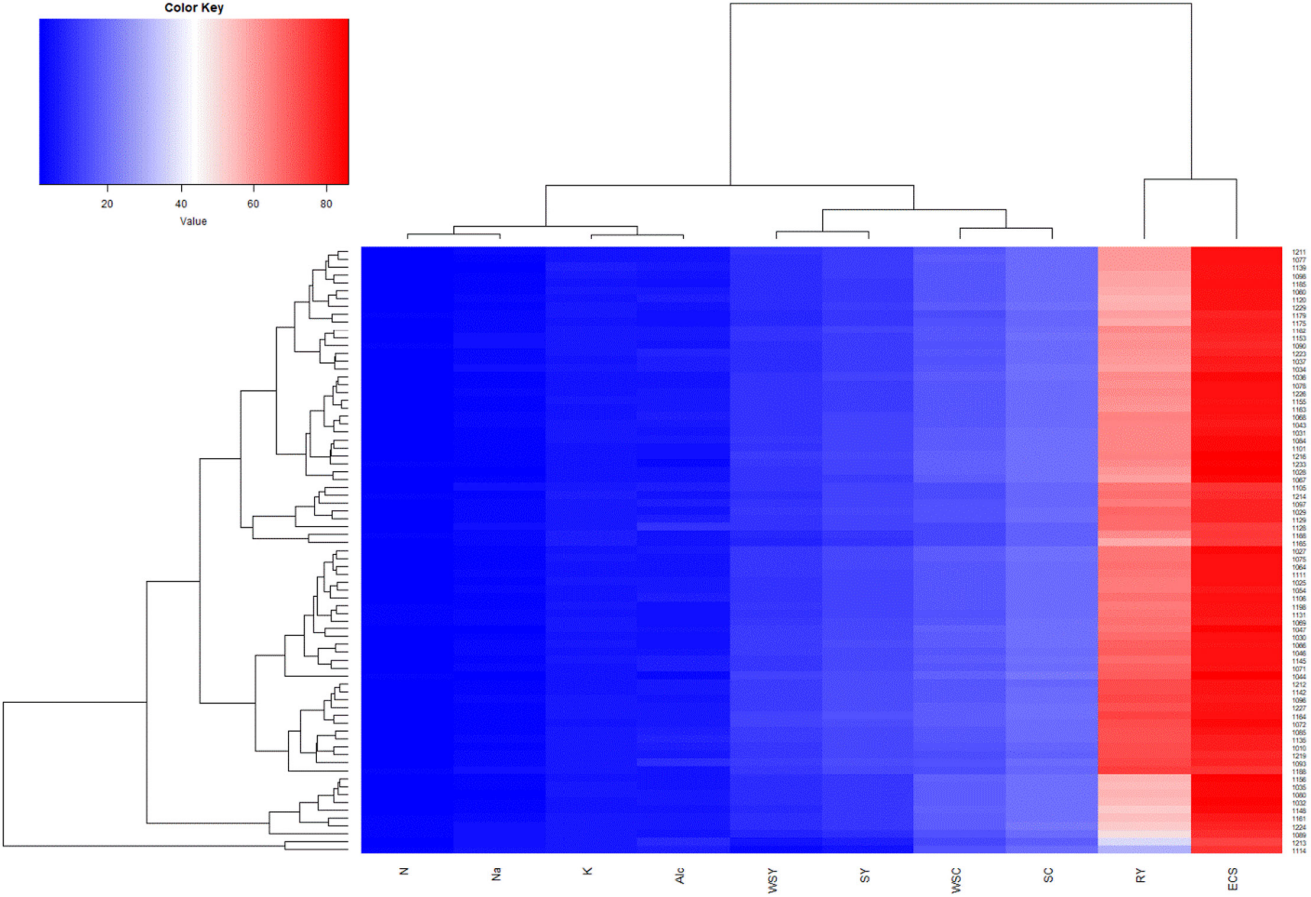
Cluster heat map analysis summarizing traits' variation of sugar beet cultivars across environments. The color intensity was adjusted according to the increase or decrease in traits. Columns and rows are clustered using Euclidean distance and complete linkage. RY, root yield; SY, sugar yield; WSY, white sugar yield; SC, sugar content; Na, sodium; K, potassium; N, nitrogen; ALC, alkalinity, WSC, white sugar content; ESC, extraction coefficient of sugar.

**FIGURE 6:**
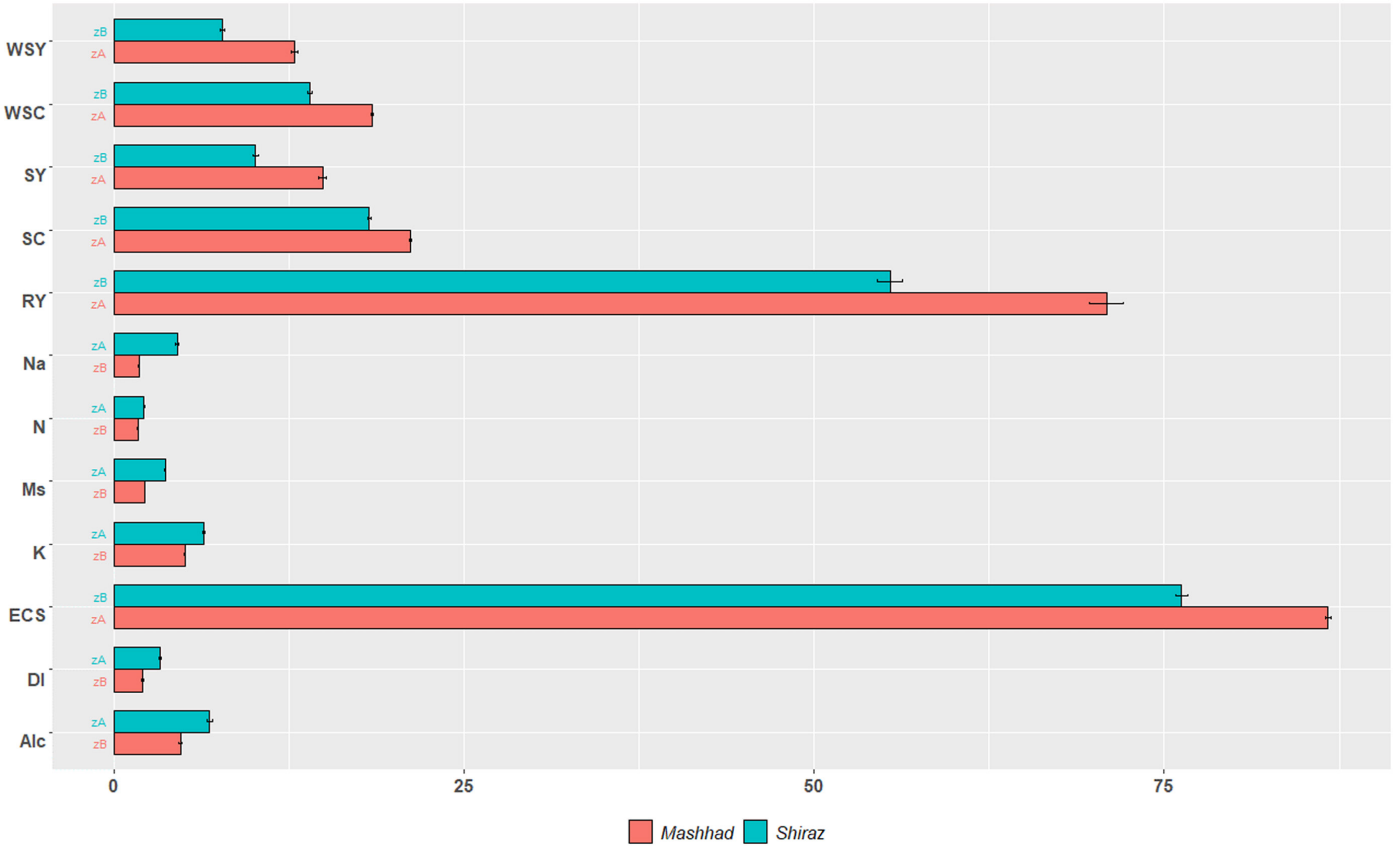
Comparison of evaluated traits among environments. RY, root yield; SY, sugar yield; WSY, white sugar yield; SC, sugar content; Na, sodium; K, potassium; N, nitrogen; ALC, alkalinity, WSC, white sugar content; ESC, extraction coefficient of sugar; MS, molasses sugar; DI, disease index.

In both regions infected with rhizomania, cultivar had a significant effect on root yield, sugar yield, sodium, potassium, amino nitrogen, extraction coefficient of sugar as well as sugar content (Figure 5). The comparison of quantitative and qualitative yields of cultivars in both regions showed that the root yield, sugar yield, sugar content, and extraction coefficient of sugar increased significantly in Mashhad region (about 1.5 times, Figure 6). In Shiraz, where the intensity of infection is higher, sodium, potassium, nitrogen, and alkalinity showed a significant increase of 2.5, 1.2, 1.2, and 1.4 times, respectively (Figure 6). The root yield and sugar yield of susceptible cultivars 1114 and 1213 were severely reduced (about 2.5 times, Figure 5). Such root yield reduction is in conformity with the similar finding made earlier by Tuitert (1994), in which root yield decreased from 70 t/ha under normal condition to 26 t/ha under high rhizomania infection. An increase in root yield could have been expected under low incidence of the disease. Sugar content has been reported to be the first parameter affected by rhizomania disease (Asher, 2002). In some trials in Shiraz, the adjusted mean of sugar content was high, which is in contrast to other studies (Henry, 1996; Kuzevski et al., 2000), and may be attributed to smaller root size and an increase in dry matter (data not shown). Muller and Gosswein (1987) cited by Tuitert (1994) evaluated the effect of irrigation on rhizomania in naturally infected fields. Their results showed that irrigation mounted sugar content. Similarly, Tuitert (1994) reported that irrigation significantly affected the linear impact of rhizomania infection; in the control and the lowest infection level, the sugar content was not influenced or increased to a slight extent. Based on the above‐mentioned studies, furrow irrigation in Shiraz could slightly decrease the influence of rhizomania on sugar content in some trials. However, the lower root yield neutralized the effect of high sugar content on final sugar yield (Figure 5).

Many studies have reported that the yield and quality of the sugar beet are predominantly conditioned by the environment and variety (Chen et al., 2021; Choluj et al., 2004; Fasahat et al., 2020; Hoffmann et al., 2009; Norouzi et al., 2017). The remarkable feature of a commercial sugar beet cultivar is producing high sugar yield, which is the result of the root yield multiplied by sugar content. The results of this study showed that rhizomania has a negative effect on both of the above‐mentioned characteristics and causes a decrease in sugar yield. Darabi et al. (2017) reported the average decrease in sugar yield for commercially resistant and susceptible cultivars as 25 and 90%, respectively. The decrease in root weight ultimately led to a decrease in extracted sugar. Henry (1996) reported 8–48% reduction in the extraction coefficient of sugar according to the intensity and the time of infection.

Sodium content can be suggested as a sensitive indicator of rhizomania, showing significant variation at low and high infection levels (1.82 in Mashhad versus 4.66 meq.100/g in Shiraz). The present results are in agreement completely with those reported by Heijbroek (1989), Tuitert (1994), Kuzevski et al. (2000), and Campbell et al. (2008) that under rhizomania infection, sodium and invert sugar accumulation increases in the root. An increase in potassium content (1.28 times) was observed with an increase in infection level, especially in Shiraz. However, in other studies, the response of cultivars to rhizomania was different. Darabi et al. (2017) reported a decrease of potassium in resistant cultivars to rhizomania, including Dorotea and Brigitta, but an increase in other cultivars. In contrast, Tuitert (1994) suggested that this parameter is less sensitive to rhizomania. According to the results, Shiraz is a favorable location for the development and establishment of virulent isolates of rhizomania disease (Rajabi et al., 2022). Because of this situation, cultivars with more effective resistance genes against the disease should be recommended for cultivation in Shiraz. Changes in root characteristics (Figure 1) decline the ability of the root to absorb water and mineral nutrients. As a result, susceptible cultivars are exposed to drought stress. This limitation of water access consequentially influences gas exchange in the plant (Keller et al., 1989).

## 4 CONCLUSION

The technological quality of sugar beet is a combination of all chemical and physical aspects that affect the production process and ultimately the amount of sugar extraction. Various factors, such as the sugar content, the rate of impurities, the percentage of syrup purity, and agronomic factors, especially the type of variety, soil texture, and climatic conditions, play a significant role in the quality of sugar beet. In this study, the effect of the most important impurities accumulated in sugar beet root, such as sodium, potassium, and amino‐N, on the technological quality of sugar beet and the sugar extraction was evaluated. This group of impurities causes a decrease in the extraction coefficient of sugar and the technological value of sugar beet and ultimately leads to an increase in molasses sugar. Results of this study showed that due to the high infection of the experimental field in Shiraz compared with Mashhad, the amount of nonsugar substances and impurities in the root was more, which is not a favorable region for the cultivation of sugar beet cultivars with common resistance to rhizomania. Considering the extensive cultivation of commercially resistant cultivars in Iran, it is noteworthy that there is a possibility of the emergence and spread of damaging virus pathotypes such as P‐type as well as resistance breaking pathotypes. Therefore, one of the most important points in the management of the disease is tracking of different populations of the virus in different regions of the country, so that if such pathotypes appear, they can be identified before they develop throughout the country. In the next step, it is necessary to evaluate the resistant cultivars under infected conditions with aggressive BNYVV pathotypes, so that superior cultivars can be identified. According to the results, variation in sodium and amino‐N can be used as an indicator for the detection of the rhizomania disease as well as for identifying infected fields and areas.

## AUTHOR CONTRIBUTIONS

**Parviz Fasahat:** Conceptualization (equal); data curation (equal); formal analysis (equal); methodology (equal); writing – original draft (equal); writing – review and editing (equal). **Mohsen Aghaeezadeh:** Conceptualization (lead); data curation (equal); resources (lead); validation (lead). **Dariush Taleghani:** Investigation (equal); supervision (equal); visualization (equal). **Mozhdeh Kakueinezhad:** Supervision (equal); writing – original draft (equal); writing – review and editing (equal). **Mostafa Hosseinpour:** Investigation (equal); writing – original draft (equal); writing – review and editing (equal). **Rosa Angela Pacheco:** Methodology (equal); software (equal); writing – original draft (equal); writing – review and editing (equal).

## CONFLICT OF INTEREST STATEMENT

The authors declare that they do not have conflict of interest. The funding body was involved in the material creation, designing the study, data analysis, and writing the manuscript.

## ETHICS STATEMENT

Not applicable.

## Supporting information


Table S1.

## Data Availability

The data that support the findings of this study are available from the corresponding author upon reasonable request.
