# Supplementary material for: Evaluation of rhizomania infection on sugar beet quality in multi‐year field assessment
Source: Food Sci Nutr. 2024 Apr 5;12(6):4100–9. doi: 10.1002/fsn3.4069 (PMC11167157; doi:10.1002/fsn3.4069)
Supplement: Supplementary file 1 — Table S1. [file FSN3-12-4100-s001.docx]

Table S1. Sugar beet cultivars used in the experiment

| Genotype code | Origin |  | Genotype code | Origin |  | Genotype code | Origin |
| --- | --- | --- | --- | --- | --- | --- | --- |
| 1010 | F-20890 |  | 1077 | F-20880 |  | 1155 | F-20933 |
| 1025 | F-21090 |  | 1078 | F-20930 |  | 1156 | F-20771 |
| 1027 | F-21177 |  | 1080 | F-20169 |  | 1161 | F-20869 |
| 1028 | F-21093 |  | 1084 | F-20940 |  | 1162 | F-20867 |
| 1029 | F-21176 |  | 1085 | F-20922 |  | 1163 | F-20672 |
| 1030 | F-21180 |  | 1089 | SBSI007 |  | 1164 | F-20188 |
| 1031 | F-21175 |  | 1090 | F-20871 |  | 1165 | SBSI031 |
| 1032 | F-21238 |  | 1093 | F-20873 |  | 1168 | SBSI038 |
| 1034 | F-21192 |  | 1096 | F-20121 |  | 1175 | SBSI045 |
| 1035 | F-20931 |  | 1097 | F-21020 |  | 1179 | SBSI049 |
| 1036 | F-20950 |  | 1098 | F-21019 |  | 1185 | SBSI058 |
| 1037 | F-21012 |  | 1101 | F-20953 |  | 1188 | SBSI068 |
| 1043 | F-20674 |  | 1105 | F-21023 |  | 1198 | SBSI133 |
| 1044 | F-20956 |  | 1106 | F-20884 |  | 1211 | F-20934 |
| 1046 | F-21029 |  | 1111 | F-20990 |  | 1212 | F-20883 |
| 1047 | F-21077 |  | 1114 | 7112 |  | 1213 | SBSI001 |
| 1054 | F-20772 |  | 1120 | F-20935 |  | 1214 | SBSI034 |
| 1060 | F-20932 |  | 1128 | F-20794 |  | 1216 | F-20989 |
| 1064 | F-21237 |  | 1129 | F-20784 |  | 1219 | F-21003 |
| 1066 | F-21174 |  | 1131 | F-20945 |  | 1223 | F-21236 |
| 1067 | F-20716 |  | 1135 | F-20704 |  | 1224 | F-20554 |
| 1068 | F-21243 |  | 1139 | F-20879 |  | 1226 | F-21244 |
| 1069 | F-21131 |  | 1142 | F-21092 |  | 1227 | F-21089 |
| 1071 | F-21172 |  | 1145 | F-20874 |  | 1229 | F-21195 |
| 1072 | F-21171 |  | 1148 | F-20707 |  | 1233 | F-20942 |
| 1075 | F-21173 |  | 1153 | F-20851 |  | - | - |
